# On Placental Toxicology Studies and Cerium Dioxide Nanoparticles

**DOI:** 10.3390/ijms222212266

**Published:** 2021-11-12

**Authors:** Gaëlle Deval, Sonja Boland, Thierry Fournier, Ioana Ferecatu

**Affiliations:** 1 Université de Paris, Inserm, UMR-S 1139, 3PHM, Faculté de Pharmacie, 75006 Paris, France; gaelle.deval@inserm.fr (G.D.); thierry.fournier@parisdescartes.fr (T.F.); 2 Université de Paris, BFA, UMR 8251, CNRS, F-75013 Paris, France; boland@univ-paris-diderot.fr

**Keywords:** nanoparticles, nanoceria, cerium dioxide, human placenta, placental barrier, toxicology studies, trophoblasts

## Abstract

The human placenta is a transient organ essential for pregnancy maintenance, fetal development and growth. It has several functions, including that of a selective barrier against pathogens and xenobiotics from maternal blood. However, some pollutants can accumulate in the placenta or pass through with possible repercussions on pregnancy outcomes. Cerium dioxide nanoparticles (CeO_2_ NPs), also termed nanoceria, are an emerging pollutant whose impact on pregnancy is starting to be defined. CeO_2_ NPs are already used in different fields for industrial and commercial applications and have even been proposed for some biomedical applications. Since 2010, nanoceria have been subject to priority monitoring by the Organization for Economic Co-operation and Development in order to assess their toxicity. This review aims to summarize the current methods and models used for toxicology studies on the placental barrier, from the basic ones to the very latest, as well as to overview the most recent knowledge of the impact of CeO_2_ NPs on human health, and more specifically during the sensitive window of pregnancy. Further research is needed to highlight the relationship between environmental exposure to CeO_2_ and placental dysfunction with its implications for pregnancy outcome.

## 1. Background

### 1.1. Human Placenta Ontogeny and Structure

The human placenta is a unique, species-specific and highly metabolically active organ. Placental physiology varies during pregnancy—ontogeny—with major modifications between early pregnancy and term. Placental ontogeny begins with the embryo’s first lineage segregation, which separates the inner cell mass from the trophectoderm. On the sixth day after fertilization, the blastocyst stage embryo encounters the “pregnant uterine endometrium”, which has, thanks to estrogen and progesterone, undergone several functional and morphological modifications called decidualization. Then, the trophectoderm will differentiate into several layers [1]:

- the villous cytotrophoblasts (VCTs), which differentiate into a syncytium called a syncytiotrophoblast (ST) by a cell-cell fusion process,
- the extravillous cytotrophoblasts (EVCTs), which invade the maternal decidua basalis up to the upper third of the myometrium, take part in the remodeling of the maternal spiral arteries and are responsible for the immune tolerance of the conceptus by expressing a non-classic human leucocyte antigen.

At the tip of the chorionic villi, the EVCTs constitute columns of proliferative cells that undergo an epithelial-mesenchyme transition, exit the cell cycle and invade the decidua basalis. This physiological invasion process occurs mostly during the first trimester and is tightly regulated since it stops at the upper third of the myometrium and is specifically oriented towards the uterine spiral arteries. They participate in uterine-placental vascularization remodeling and form trophoblastic plugs within the terminal section of the arteries, thus preventing the flow of oxygenated maternal blood into the intervillous chamber during the first 10 weeks of amenorrhea (WA). Therefore, the early development of the placenta occurs under physiological hypoxia (pO_2_ of 20 mmHg). Between 10 and 14 WA, the gradual disintegration of the trophoblastic plugs results in the arrival in the intervillous chamber of oxygenated maternal blood, with a partial pressure of oxygen of 60 mmHg until the end of the pregnancy [2].

**Figure 1:**
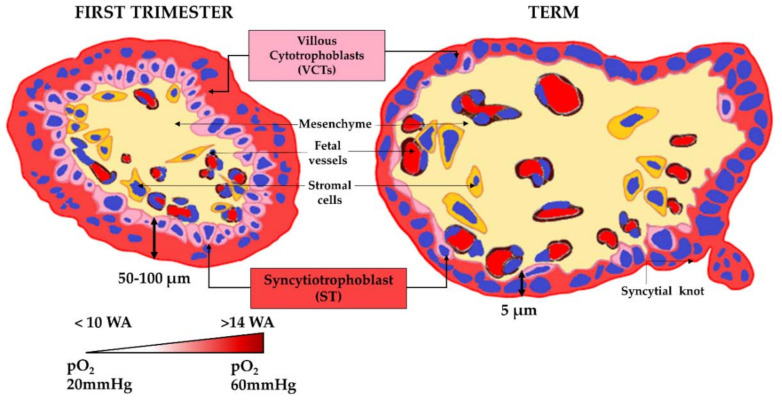
Diagram of a cross-section of a villus in the first trimester of pregnancy and at term. The placental barrier is made of syncytiotrophoblast (ST) and villous cytotrophoblast (VCT). The thickness of this barrier decreases during pregnancy from 50–100 µm at the first trimester to 5 µm at term. The constant renewal of the ST occurs by fusion with the VCTs and the release of syncytial knots. In the center of the chorionic villus, the fetal vessels are surrounded by mesenchyma and stromal cells. The oxygen pressure in the intervillous chamber varies during the first trimester from 20 mmHg (2–3% O_2_) before 10 WA to 60 mmHg (6–8% O_2_) above 14 WA.

At the term of pregnancy, the human placenta is a discoid organ measuring around 20 cm in diameter. The ratio of placenta to fetal weight is reversed during pregnancy to give a term placenta weighing approximately 500 g, or 1/6th of the fetal weight. Considered as the most invasive type of placentation as compared to other species [3], human placentation is highly specific and characterized as hemochorial, because the chorion is directly immersed in the maternal blood within the intervillous chamber. The structural and functional unit of the human placenta is the chorionic villous (Figure 1), which is composed of a fetal mesenchymal axis embedding the fetal vessels and surrounded by an epithelial layer of trophoblasts.

On the fetal side, the trophoblast layer is composed of mononucleated VCTs, bordered by a multinucleated ST on the maternal side. VTCs, which are based on a lamina, are partially differentiated cells harboring special properties. For instance, most of them have exited the cell cycle and are thus non-proliferative except for a small number of progenitor cells. Their terminal differentiation into ST occurs after a cell fusion process. However, genetic diseases (such as trisomy 21 [4]) or exposure to some compounds (delta-9-tetrahydrocannabinol [5,6], at certain low doses, mono(2-ethylhexyl) phthalate (MEHP) [7]) can alter the capacity of the trophoblast to differentiate terminally and form the ST. In contrast, other pollutants, such as benzo-(a)-pyrene and bisphenol A, increase the syncytialization of the trophoblasts [8,9]. ST is a multinucleated and polarized syncytium, with rich microvilli on the apical side (directly bathing in the maternal blood), thereby increasing the exchange surface area with the maternal bloodstream. Throughout pregnancy, ST is constantly renewed by the differentiation and fusion of the underlying VCTs with the existing ST, and by the release into the maternal circulation of syncytial fragments, called knots [10]. These syncytial knots contain aging organelles and apoptotic nuclei. Their production is increased in the case of pre-eclampsia [11]. Pre-eclampsia, which is a pregnancy pathology specific to humans, originates from the defective invasion of the endometrium by the extravillous trophoblast leading to insufficient remodeling of the uterine arteries, hypoperfusion of the placenta and ST dysfunction. This pathology involves oxidative stress and inflammation leading to generalized dysfunction of the ST and causes the release of necrotic syncytial fragments and antiangiogenic factors into the maternal circulation, which is responsible for generalized maternal vasculopathy. This placental pathology remains a major cause of maternal and fetal morbidity, mortality and prematurity.

The role of the ST is essential since it constitutes the first contact tissue with the maternal blood and is the seat of placental functions. The ST ensures the synthesis and secretion of a panoply of hormones, such as steroids, glycoproteins and indispensable factors for fetal development and growth and for maternal adaptation to pregnancy. The ST is also an exchange tissue that ensures nutrition by diffusion and transporters. The ST is the first barrier against xenobiotics and protects the fetus against xenobiotics to which the mother may be exposed. Many substances can, however, cross the placenta, by using transporters, for instance.

### 1.2. Human Placental Functions

The first role of the placenta is embryo implantation into the maternal endometrium. Subsequently, it allows maintenance of the pregnancy, and development and growth of the embryo. One of its main roles is exchanging nutrients (amino acids, fatty acids, glucose, etc.) and gases (O_2_, CO_2_) between the maternal and fetal circulations as well as the evacuation of fetal waste products.

The placenta is an endocrine organ [12] able to synthesize and secrete several hormones such as human chorionic gonadotropin (hCG), human placental lactogen (hPL) and steroid hormones (progesterone and estrogens, such as estrone, estradiol and estriol), as well as factors such as the placental growth factor (PlGF) and soluble fms-like tyrosine kinase-1 (sFlt1). As said before, this function is mainly assured by the ST. These hormones and factors play important roles in fetal development and growth, adaptation of the maternal organism to pregnancy (including immunological tolerance) and parturition. Furthermore, the endocrine function differentiates the human placenta from other species such as rodent placenta.

### 1.3. The Placental Barrier

While the maternal body is constantly exposed to xenobiotics, the placenta acts as a selective barrier. Some substances are able either to accumulate in and/or be metabolized by the placenta. Others even cross the placental barrier to the fetal side by passive diffusion (in the case of molecules of low molecular weight, non-ionized and lipophilic), by means of active transporters [13] or by different types of endocytosis (pinocytosis, phagocytosis, receptor-mediated endocytosis) [14]. Different parameters can influence placental accumulation or passage of a xenobiotic: its characteristics (molecular weight, ionization, liposolubility), placental factors (physiological variations in the placental barrier between the first trimester and term placenta) and finally maternal and fetal factors (levels of proteins such as albumin which can bind to xenobiotics, blood pH, vascularity, metabolism).

The human placental barrier is mainly formed by the epithelial bilayer of trophoblasts (VCTs and ST) on the surface of the villi. However, the thickness and size of this barrier, as well as the contact with oxygenated maternal blood, vary as pregnancy advances in different ways, such as:

- an increase in the exchange surface area through the continuous ramifications of the villus tree to reach an area of 14 to 20 m^2^ at term;
- a decrease in the trophoblastic bilayer width, and therefore in the epithelial barrier between the maternal and fetal bloodstreams, dropping from 50 µm in the second month of pregnancy to 5 µm at the end of pregnancy;
- the arrival of maternal oxygenated blood in the intervillous chamber in contact with the ST between 10 and 14 WA after the removal of the trophoblastic plugs;
- an increase in the uterine blood flow up to 600 mL/min at the term of pregnancy.
- All these physiological changes must be considered when studying the effects of pollutants on the placental barrier and throughout placental ontogeny.

### 1.4. Impacts of Pollutants on the Human Placenta

Maternal blood can contain many pathogens and xenobiotics, such as viruses, addictive substances internalized consciously by pregnant women (drugs, alcohol, tobacco, etc.) or unconsciously (pollutants produced by human activities, such as ultrafine particles in the atmosphere (UFPs), polycyclic aromatic hydrocarbons, nanoplastics, etc.). Placental internalization of some xenobiotics may alter the normal course of pregnancy and fetal development. However, the harmful effects of pollutants on the fetus do not only depend on the ability of the pollutants to cross the placental barrier, from the maternal blood flow to the fetal side. Their direct impact on the placenta by accumulation, biopersistence and metabolization by this key organ can be just as deleterious for pregnancy. In addition, as previously explained, the permeability of the placental barrier varies during pregnancy. The consequences of exposure in the first trimester and at the end of pregnancy can often differ, with more severe deleterious effects during the first trimester when placentation and organogenesis of the fetus occur.

A well-known example of xenobiotic impacts on pregnancy is tobacco consumption. Smoking during pregnancy is associated with intrauterine growth retardation (IUGR), an increased risk of bleeding with placental abruption and placenta previa [15], miscarriage [16] and preterm delivery. These pregnancy pathologies caused by smoking result from placental dysfunctions such as an alteration of nutrient transporter expression [17], a decrease in the morphological and functional differentiation capacity of trophoblasts, the disruption of angiogenic factors [18] (sFlt1 and/or PlGF), a decrease in the intervillous space [19] and epigenetic alterations of DNA by methylation [20].

Other impacts of xenobiotics on pregnancy include fetal alcohol syndrome due to alcohol consumption and teratogenic effects due to some pharmaceutical drugs. The major teratogenic effects of thalidomide have been known since 1961 [21]. The long-term consequences for reproduction in women exposed in utero to distilbène were described since the 1970′s [22]. In 1986, Barker and Osmond developed the concept of the developmental origins of health and disease (DOHaD) [23] and, in 1993, Barker et al. established for the first time the link between a fetal origin and the development of a disease in adulthood [24].

Nowadays, the impact of pharmaceutical drugs during pregnancy is well studied, and a precautionary principle prevails when prescribing treatment during this period. Health professionals educate pregnant women about the risks of consuming certain substances such as alcohol and tobacco. However, the impact of pollutants during pregnancy is less well known by the general public, as new human-made pollutants emerge in the environment. In recent years, awareness developed regarding the harmful impact of certain pollutants released in our environment by human activities or found in consumer products to which pregnant women are also exposed, such as bisphenol A [25] or phthalates [7], with the emergence of the concept of endocrine disruptors [26].

The placental internalization of some pollutants can alter the maintenance and physiological development of the placenta, especially during early exposure within the first trimester of pregnancy, which can then impact the development or growth of the fetus [27]. Pollutants can also lead to changes in placental epigenetics [28] and affect pregnancy outcomes [29,30]. In agreement with the DOHaD concept, their impact may extend beyond the prenatal period with neurodevelopmental impairment in children exposed in utero [31,32], and predispositions to some pathologies in adulthood, such as type II diabetes [33] and cardiovascular diseases [34]. Transgenerational effects were also observed in rodents following exposure to certain pollutants, such as dioxin. Impacts on the fertility of subsequent generations as well as on the male/female ratio of litters are reported [35].

The impact of emerging pollutants such as nanoparticles (nano-objects whose three dimensions are less than 100 nm) on the placental barrier is still poorly understood, especially as their impact can vary depending on multiple parameters such as nanoparticle shape, size, surface charge, agglomeration/aggregation and chemical composition [36,37]. This is the case with cerium dioxide nanoparticles (CeO_2_ NPs), a new pollutant already found in ambient pollution including from automobile traffic exhaust and cigarette smoke. In addition, humans are rarely exposed to just one type of nanoparticle from ambient pollution, such as CeO_2_ NPs. The study of such exposure should take into account the possible cocktail effect with other pollutants emitted simultaneously by the same sources of pollution. Indeed, exposure to mixtures of pollutants is closer to environmental reality. Interactions between different pollutants can modify their properties, their biopersistence and modulate their toxicity.

## 2. Strategies to Study the Impact of Pollutants on the Placenta Barrier

Different models are now available to study the placental barrier, each with advantages and limitations. Each model provides answers to specific questions (Table 1). Although there is no perfect model, similar results when combining several of these models for toxicology studies give a clearer vision of their effects.

### 2.1. Animal Models

First, in vivo studies on small animal models, mainly rodents, have provided many advances regarding the impact of pollutants during gestation, especially during different periods of pregnancy. Rabbits, mice and rats, similar to humans, have a discoid and hemochorial placenta, and their low cost makes them more attractive for study than large primates, which also have hemochorial placentation. In attempts to understand the transplacental passage of pollutants and their accumulation in the fetal compartment, such animal models are indispensable for the evaluation of fetotoxicity, which cannot be tested directly in humans for obvious ethical reasons. However, caution should be exercised in the extrapolation of data from animal models to humans, given the high specificity of the human placenta, as there are several differences to take into account, such as [38,39]:

- hemotrichorial placenta in rodents, composed of three trophoblast layers (one of VCT and two ST) instead of a trophoblast bilayer in humans (VCT and ST);
- the human placenta has several cotyledons on the maternal side of the placenta, unlike placenta in rodents;
- a labyrinthine organization (resulting from the fusion of villi around maternal blood gaps) in rodents;
- lack of hCG and of steroid hormone production by rodent placenta (e.g., steroids are secreted by the ovary during gestation);
- a more superficial invasion of maternal decidua in mice;
- the period of gestation (19–20 days for mice versus 270 days for humans).

However, there are multiple similarities between human and rodent placenta, such as the deep invasion of trophoblasts. 

### 2.2. Ex Vivo Placental Perfusion

Placental perfusion provides data on the transplacental passage of certain xenobiotics, their accumulation and metabolism in tissues, as well as their possible effects on placental structure [34,35]. This technique, developed by Panigel in 1967 [40] and then improved by Schneider in 1972 [41], is based on the principle of reproducing the maternal-fetal circulation for a cotyledon. The veins and arteries of the umbilical cord as well as the intervillous chambers are catheterized within one hour of placenta delivery. Placental perfusion, which can be maintained for up to 6 h, forms an open circuit when the maternal and fetal perfusates do not circulate in a loop, allowing the kinetics of a xenobiotic to be studied. It is also possible to create a closed circulation system allowing the study of placental metabolism. In order to ensure the tissue integrity of the perfused cotyledon and to standardize the results, a marker such as antipyrine, which crosses the cotyledon by passive diffusion only, is added to the maternal circulation at a limited rate. Since interindividual variations can be significant from one placenta to another, several cotyledons perfused from different placentas are necessary to normalize the results. After the perfusion, the villi can be recovered from the perfused cotyledon to observe the accumulation and metabolization of the xenobiotic of interest. This model is to date the most physiological for the study of the placental barrier, but has certain limitations. Technical problems such as intervillous chamber leaks or anatomical variations (cotyledon perfused by a different vessel than the one catheterized) can occur, which lowers the method’s success rate to approximately 50% [42]. In addition, the current technique is only possible for placentas in terms of pregnancy and does not allow the study of chronic exposure.

### 2.3. Chorionic Villous Explant Cultures

**Figure 2:**
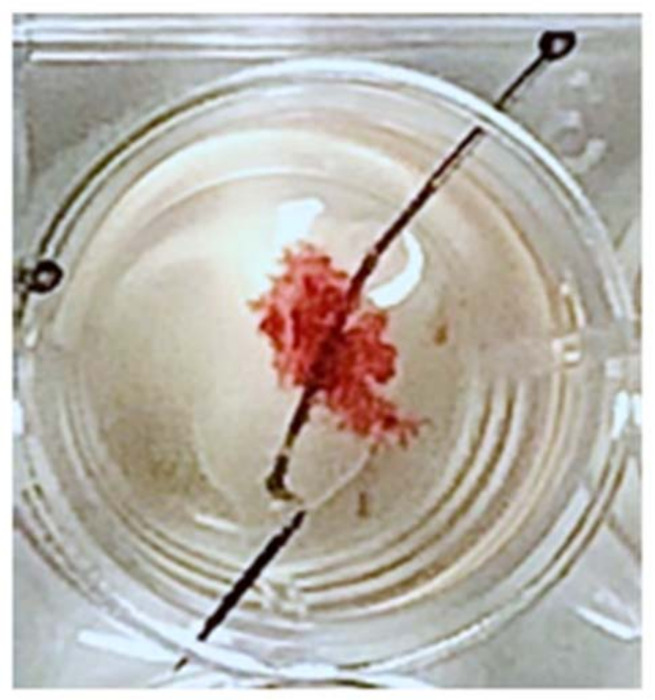
Chorionic villous explants on a needle. Chorionic villous explants are obtained after dissection of a human placenta at term or from the first trimester of pregnancy. The villous explants are kept in suspension in the culture medium as hanging villi after having been threaded on a needle.

Direct use of human placenta, which is easily accessible after childbirth or the termination of pregnancy, allows toxicology studies both at term and in the first trimester of pregnancy. A physiological model for toxicological studies is the culture of villous explants in which all types of placental cells are represented while preserving the tissue architecture. After placenta dissection, fragments of chorionic villi can be recovered and are incubated either in suspension as hanging villi, after mounting them on a needle [43,44] (Figure 2) or on a matrix (Matrigel or type I collagen). Matrigel is a reconstituted basement membrane secreted by Engelbreth-Holm-Swarm (EHS) mouse sarcoma cells which contains all the elements of an extracellular matrix: glycoproteins (laminin), fibers (collagen), heparan sulfate proteoglycans and growth factors (TGF-β, EGF).

Chorionic villous explants, which produce hormones that can be assayed in the supernatant, can be maintained for up to 12–15 days. The ST, though, rapidly degenerates after 24 h, and a new ST is then formed [45]. In toxicology, this model is easy to set up and to use and thus to study the impact of a xenobiotic present in the intervillous chamber on the chorionic villi.

### 2.4. Primary Culture of Trophoblasts (EVCT, VCT and ST)

In 1986, Kliman et al. first cultivated human primary trophoblasts using a protocol to isolate VCTs from term placentas. This protocol includes the digestion of the placental villi with a trypsin-based solution followed by the isolation of the VCTs using a Percoll gradient [46]. Primary cultures of VCTs, derived from term or first-trimester placenta, retain their in vivo functions regarding hormone secretion and their spontaneous differentiation capacity to form the ST. Primary VCTs are non-proliferative and are therefore closer to physiological reality, but they are fragile cells. Indeed, ST formed by VCTs dies by spontaneous necrosis beyond 5 days of culture, which makes VCTs unsuitable for studying the impact of long-term chronic exposure. Another great advantage is that VCTs can be isolated from both term and first-trimester placenta (although not 100% efficient for the latter), allowing a wide window to extrapolate the toxicity during pregnancy. The only limits are general to all in vitro cellular models when they are cultivated under 21% O_2_ and in Petri dishes, which are hugely different from the placental architecture and physiological cell microenvironment. Changes in their microenvironment and lack of tissue interaction can cause them to behave differently.

### 2.5. Trophoblast Cell Lines

The placental models most employed to study placental toxicology are human trophoblast cell lines. Several cell lines were generated since 1968 from trophoblast-derived tumors (choriocarcinomas), such as BeWo [47], JEG-3 [48] and JAR [49], and are still widely used today. BeWo lines are employed as a model of the villous cytotrophoblast mostly for their ability to differentiate and form a syncytium (reminiscent of VCT) after forskolin treatment (a cyclic AMP activator) and to produce all placental hormones including hCG. Thereafter, immortalized placental cells lines as HTR-8, HTR-8/SVneo [50] and human invasive proliferative extravillous cytotrophoblast (HIPEC 65) [51] were derived from VCTs transformed by infection with simian virus 40 (large tumor antigen), by adenovirus or by human papilloma virus. Trophoblastic lines are an easy, fast and inexpensive way to study placental toxicity, and thus they are the most widely used models in placental toxicology. However, their cancer/immortalized cell qualities (robustness and ease to grow) are also their main flaw. Their mutations distinguish them from physiological placental cells and firmly established the inhibition of the p53 and retinoblastoma (Rb) family of tumor suppressors leading to unlimited proliferation. Cell lines acquire different DNA methylation profiles as well as variations in physiological parameters [52,53] and membrane transporter expression [54]. In contrast to cell lines, placenta purified VCTs in a primary culture spontaneously aggregate and fuse to form the ST, after 48 to 72 h, unlike BeWo lines whose fusion must be induced by forskolin, an activator of PKA (via cyclic AMP). Moreover, VCTs are partially differentiated cells intended to merge into ST and are non-proliferative in vitro, unlike trophoblast cell lines, which are immortalized and proliferative and thus behave differently. For instance, some trophoblast lines have lost contact inhibition and can form multilayer structures unlike physiological VCTs. Thus, when employing cell lines as a toxicity model, it should be taken into account that such cells are prone to resist apoptosis induction. As previously described for primary trophoblast cultures, cell line cultures are limited by a microenvironment that differs from physiological conditions. In order to overcome the 2D cell monoculture limitation, the recent development of 3D in vitro reconstruction models, including of the placental barrier, allows a closer to physiological evaluation of the impact of some pollutants on the placental barrier.

### 2.6. Co-Cultures and 2.5D Two-Chamber Models 

The sandwich culture is a 2D model of a co-culture using 2 or 3 cell types (trophoblasts, fibroblast and endothelial cells). The different layers of cells can be separated by a membrane (amniotic membrane [55], Transwell insert with microporous membrane [56]) within a two-chamber model, which can itself contain a cell type (methacrylated gelatin membrane containing fibroblasts [57]), or on the contrary several cell types can be mixed in the same layer thanks to nanofilm technology allowing cells to be coated with fibronectin and gelatin [58].

In recent years, Transwell inserts have been increasingly used for co-culture because of their ease of implementation and high experimental success rate. The cell lines separated by a porous membrane of variable size will form an epithelium whose transepithelial electrical resistance can be measured with a voltmeter. The cells can also secrete different molecules and proteins in the two compartments and thus are polarized with a basal side and an apical side that can be treated with a xenobiotic of interest. The impact on the tightness of the epithelium, the passage from one compartment to the other and accumulation of molecules in the basal compartment can be analyzed. 

This model also allows observation of the migration of cells through the pores of the inserts, whose size can be chosen, such as the migration of EVCTs during the first trimester of pregnancy. The culture of primary EVCTs is also possible with Matrigel, allowing them to keep their invasion capacity and to be maintained in culture for at least a week [59].

However, the models proposed only use cell lines because the primary VCTs are not proliferative, and so it is difficult to form a contiguous epithelium. Besides, the trophoblast layer often composed of BeWo cells differs from the physiological trophoblast bilayer (ST and VCT). The use of trophoblast stem cells grown under differentiation conditions to form the ST layer on the Transwell insertion membrane is currently under development [60].

### 2.7. Placenta-on-a-Chip Models

The placenta-on-a-chip technique—which is considered as a 2.5D model—combines the cell culture in a two-chamber model with microfluidics allowing continuous perfusion of both chambers with the media. This model allows the reconstitution of the placental barrier by the co-culture of 2 cell types (trophoblasts on the maternal side and endothelial cells on the fetal side) separated by a porous membrane. Each compartment is connected to microchannels allowing maternal and fetal flows [61,62,63,64]. This model reproduces the cell architecture, mechanical constraints and key placental functions, with a diffusion of factors and secretion of hormones. However, this model is still under development and is not yet available on the market.

However, because of the fragility and lack of proliferation of primary cells, most of these co-cultures use cell lines rather than primary cells, which again raises the issue of the extrapolation of cell lines to physiological conditions. Today, only one 3D model using primary VCTs is available to overcome this limitation [58] (Table 2).

### 2.8. 3-D Models

The models that most mimic the cellular microenvironment are the 3D models with spheroids and organoids (Table 3). The 3D spheroids can be easily obtained using a hanging drop system [66] allowing a co-culture with a fibroblastic core corresponding to the fetal side and a layer of trophoblasts all around for the maternal side.

Human trophoblast organoids develop as 3D structures anatomically and functionally close to the in vivo villi [67,68]. Cell clusters that express a marker of proliferative trophoblasts isolated from first-trimester placentas are seeded into Matrigel drops and grown in a basal trophoblast organoid medium containing different growth factors and signaling inhibitors, previously shown to promote the stemness and formation of organoids. Trophoblast organoids can be established in 2 to 3 weeks, after at least 2 passages. The proliferative trophoblasts can differentiate into both ST and EVCTs. The 3D organoid culture system could allow the study of the impact of pollutants on EVCT differentiation, which was not attainable with other culture models. In addition, this model is a genetically stable long-term culture that could permit the study of chronic exposure. Yet, there is a major flaw in the current technique: the polarity of the organoids. Indeed, the cells orientate themselves with their basal surface in contact with Matrigel and the apical surface where the ST differentiates towards the center of the organoid structure. Therefore, the current architecture of these organoids does not allow toxicological analysis since the pollutants would not be in first direct contact with the ST. If future improvements allow the polarity of organoids to be reversed, this model could be truly relevant for the study of the placental barrier.

## 3. Current Knowledge on Nanoceria

### 3.1. Introduction to Nanoparticles

Nanoparticles (NPs) are nanomaterials with an aerodynamic diameter of between 1 and 100 nm in all 3 dimensions (ISO/TS 80004:2015), which is the size of a virus. NPs are used increasingly in many fields (additives in cosmetics, food, food packaging, fuels and cigarettes but also as potential therapeutic drugs) due to the specific physiochemical properties that the nanoscale confers, in particular their high surface reactivity. For example, titanium dioxide nanoparticles (TiO_2_), which are among the most widespread nanoparticles, with interesting properties, are thus used as whitening, anti-corrosion and photocatalytic agents. They are widely used in many fields: as additives in food (additive E 171) and in cosmetics (including toothpaste and sunscreens), as pigments in paint, plastic and ink [69]. The introduction of these nanoparticles into the human environment raises the question of their impact on human health. The toxicity of NPs may vary greatly by distinct molecular mechanisms [70] depending on several parameters such as the chemical composition, state of agglomeration/aggregation, oxidative status for metallic NPs, shape (spherical, cubic, ovoid. etc.), surface charge (zeta potential) and surface modification. The type of exposure (acute vs. chronic), concentration and the cells or tissue model exposed to these NPs also influence their toxicity. Unlike other pollutants, such as polycyclic aromatic hydrocarbon, NPs are not metabolized by the classical xenobiotic aryl hydrocarbon receptor (AhR) detoxification pathway. Therefore, NPs tend to persist within the organism, in the plasma [71] and accumulate in exposed cells. Within the cell, they interfere with or activate specific signaling pathways [72], act as genotoxicans by direct interaction with DNA, or show effects indirectly by modification of the redox balance resulting in the production of reactive oxygen species (ROS) leading to damage to DNA or altering the DNA repair mechanisms. In addition, sustained ROS production can induce inflammation and cytotoxicity. Moreover, their great surface reactivity gives them the ability to interact with and bind other molecules to form a corona which can modify their cellular internalization, biopersistence and kinetics in the body [73].

Currently, there is no international standard regulation regarding the production, handling or labeling of NPs. Regulators such as the United States Environmental Protection Agency in the U.S. and the Health and Consumer Protection Directorate of the European Commission have started to assess the risks of nanomaterials, giving rise to state-dependent regulations. In France, for instance, the Grenelle II law (articles L. 523-1 to L. 523-3 of the environmental code), which was enacted on 1 January 2013, requires manufacturers, importers and distributors to declare each year the quantities and uses of NPs as soon as the threshold exceeds 100 g per year and per substance. Since 2017, all food ingredients present in the form of manufactured nanomaterials are clearly indicated in the list of ingredients by the word (nano). In addition, use of the additive E 171 was suspended in France on 1 January 2020. European regulations require the labeling of products containing nanomaterials for food, cosmetics and biocidal products, but these regulations are poorly applied.

NPs can also be released in the ambient air by exhaust gases or industrial activities and form an integral part of air pollution. They are then called ultrafine particles or PM_0.1_ (particulate matter with a diameter of less than 0.1 µm). For now, there are no regulations in France concerning them, unlike for PM_10_ and PM_2.5_, whereas UFPs can be even more harmful than larger particles because they penetrate deeper into the respiratory system in the alveolar region [74] and in the body [75]. Their impact on human health still needs to be explored [76] because of the lack of scientific knowledge and the absence of evidence demonstrating the safety of certain nanotechnology products make regulation very difficult. However, the French organization Airparif, which monitors air quality in the Paris region, set up a device in September 2019 to count particles in 256 size classes using an electric mobility particle size spectrometer. The particle count is a relevant parameter because although the mass of these NPs is negligible compared to that of all other particles in the ambient air, they nevertheless represent 80% to 87% of the total number of particles. In addition, human exposure to ambient air pollution rarely involves a single type of NP, but instead NPs often associate with other pollutants from the same or different sources to form complex mixtures.

### 3.2. Impact of Nanoparticles during Pregnancy

Biomedical applications of NPs are wide-ranging, mainly in oncology therapies [77]. The use of nanotherapy in pregnant women is being studied [78], considering that the treatments used must not affect the proper functioning of the placenta and the development of the fetus. Therefore, data on the bypassing of the placental barrier by NPs obtained in part by ex vivo cotyledon perfusion are essential for the development of these therapies [79]. Several air pollution NPs have been detected in human placentas [80] and some of them, such as carbon black, TiO_2_ and silver, are known to cross the human placental barrier [81,82]. However, the risks of NPs for human pregnancy are not only limited to the crossing or not of NPs through the placental barrier. In fact, indirect effects of NPs on placental growth, the production of placental hormones or on oxidative stress can also impact fetal development [83].

### 3.3. Nanoceria Properties

Cerium is a metal that belongs to the lanthanide group of ceric rare earths. It oxidizes rapidly on contact with air. Cerium dioxide (CeO_2_) NPs are industrially manufactured mainly by the precipitation method, the hydrothermal method, green synthesis, the microwave-assisted method, the micro-emulsion method, the oxidation method and sonochemical synthesis [84]. Because of their high catalytic properties, nanoceria have been added to Diesel fuel (as additives such as Envirox, a fuel borne catalyst based on nanotechnology) to increase the efficiency of soot combustion during the regeneration process of particulate filters used to reduce particulate matter emissions and increase fuel efficiency. The nanoceria are therefore released into the ambient air by the exhaust gases of these cars, with a Cerium (Ce) content increased by 6.5% [85]. However, their addition to diesel gasoline increases by a 35% emission of benzo-(a)-pyrene [86], which is known to be carcinogenic, mutagenic and reprotoxic. The addition of CeO_2_ NPs to diesel gasoline could, however, reduce some harmful effects caused by diesel exhaust such as the decrease in AP-1 (an oxidative stress-responsive transcription factor) in the brain [87]. Due to these catalytic properties, nanoceria have also been added to cigarettes since 2004 [88], self-cleaning ovens and polishing solutions [89]. One of the main sources of cerium in indoor air is cigarette smoke [90,91]. In contrast to TiO_2_ NPs to which humans are mostly exposed orally, the human exposure to CeO_2_ NPs is mainly through inhalation and not ingestion, except for their use as potential drugs or drug delivery systems.

In 2010, the Organization for Economic Co-operation and Development put CeO_2_ NPs on the list of priority nanomaterials requiring urgent evaluation because little is known of their impact on human health [92]. Several studies investigated the consequences of exposure to CeO_2_ NPs, particularly on the lungs and the immune system, with often conflicting results regarding their cytotoxicity and their pro- or antioxidant effects. Indeed, cerium has two oxidation states in the lattice structure: Ce^3+^ and Ce^4+^. Oxygen vacancies or defects in the lattice promote the Ce^3+^ reduced state. Thus, nanoceria have the ability to act as a catalyst for both oxidation and reduction reactions, mimicking the activity of different enzymes such as superoxide dismutase (SOD), catalase and peroxidase [93]. Anti- and pro-oxidant effects are observed depending on the Ce^3+^ or Ce^4+^ surface concentrations, pH, H_2_O_2_ and chelating ligand concentrations (Table 4).

Thanks to their properties, CeO_2_ NPs (also called nanoceria) are currently being studied for use in several therapies [94,95]:

- As an antioxidant for therapies designed to reduce oxidative stress in many fields: neurology, ophthalmology [96], hepatology [97], cardiology [98], fertility [99] obesity [100,101], and even space [102] to fight the oxidative stress caused by the decrease in gravity for several days or weeks;
- As a pro-oxidant, in particular by the Fenton reaction, to kill cancer cells [103]
- As a carrier for targeted drug and gene delivery thanks to their coating ability and pH-dependent oxidation state, mainly in oncology therapies [104]
- As an antibacterial against Gram-positive and Gram-negative bacteria [105]
- As an anti-diabetic and anti-obesity drug due to its superoxide dismutase 1 mimetic and anti-apoptotic activity [100,101]
- In regenerative medicine and tissue engineering by enhancing long-term cell survival, enabling cell migration and proliferation and promoting stem cell differentiation [106].

### 3.4. Nanoceria and Human Health

Most studies of the impacts of nanoceria on human health are conducted on rodents or using human cell lines. The potential toxicity of nanoceria depends on the physicochemical properties of the CeO_2_ NPs used in the study [107], the cell/tissue context, the doses and time of exposure. This may explain, in some instances, the conflicting results from one study to another. Most of the studies focus on the impacts of nanoceria on biological barriers (pulmonary, blood-brain, intestinal) and on the mononuclear phagocyte system (in the liver and the spleen [108]) where 90% to 95% of the nanoceria present in the blood will accumulate and persist for months. Indeed, the clearance of CeO_2_ NPs inhaled and distributed in the body (lungs, spleen, liver, kidney) in rats is insignificant 48 h to 72 h after exposure, which may suggest retention of NPs in the organs [109]. Less than 1% of the dose deposited in the lungs is estimated to cross the pulmonary barrier, and the percentage is even lower for the intestinal barrier [110]. Very low levels of CeO_2_ NPs may cross the cutaneous barrier in the presence of cutaneous lesions, with little dermal absorption and transdermal permeation of cerium [111]. Cytotoxicity and genotoxicity are observed in some cell types. The known mechanisms of action inducing toxicity mainly involve oxidative stress.

Although poorly studied, the formation of a protein corona on nanoceria in biological fluids could modify their effects. Regarding the possible constitution of a corona in human blood, CeO_2_ NPs were found to have more affinity for fibrinogen than for human serum albumin [112]. In this context, it is important to note that unfolding of fibrinogen due to binding to NPs has been proposed as an alternative mechanism of NP-induced inflammation [113]. The formation of a protein corona could also have a protective effect by, for instance, allowing internalization of nanoceria via clathrin-mediated endocytosis, thereby preventing cytotoxicity induced by plasma membrane disruption. Clathrin-mediated endocytosis appears to be due to the interaction of transferrin present in the protein corona with the transferrin receptor [114].

The pathways of the cellular internalization of CeO_2_ NPs are multiple and dependent not only on the protein corona, but also on the physicochemical parameters of the particles. As mentioned previously, Clathrin-mediated endocytosis but also caveolae-mediated endocytosis were reported [115] and even passive uptake of small CeO_2_ NPs (3–5 nm) was observed [116].

The identification of the various factors involved in the toxicity of CeO_2_ NPs as well as the understanding of their mechanisms of action are not only important for risk assessment but could also allow the design of safer nanoceria in the future, mainly for biomedical applications.

### 3.5. CeO_2_ and Pregnancy

Data on the impact of CeO_2_ NPs during pregnancy are very scarce, and, to date, only one study used human placental physiological models [117]. Cerium was found in breast milk and blood samples from mothers in cohorts of pregnant women in Germany and Spain [118]. Blood cerium concentrations (mean 10 ng/L up to 70.3 ng/L) differed significantly depending on the women’s city of residence. A correlation was thus established between the cerium concentrations and the environmental pollution corresponding to each city. To date, the ability or not of CeO_2_ NPs to cross the placental barrier has not been evaluated by the ex vivo placental perfusion technique. The transfer of radiolabeled cerium from mother to fetus in rats was determined at 0.05% of the injected dose [119]. However, a few relationships were observed between exposure to nanoceria during gestation and observations on newborns. A recent epidemiological study showed a correlation between high levels of cerium in maternal blood (higher than 78 ng/L) and abnormalities in the closure of the neural tube in newborns [120]. Furthermore, increased cerium levels in maternal urine (60 ng of cerium/g of creatinine) were associated with decreased neonatal TSH levels in a cohort of 7300 women in China [121].

As for the impact of nanoceria on human reproduction, data on their impact on pregnancy in rodents differ from one study to another (Table 5).

In mice, intravenous exposure (5 mg/kg for 3 days) to CeO_2_ NPs in maternal blood in early pregnancy leads to adverse pregnancy outcomes with severe placental dysfunctions (altered decidualization with the disruption of decidual cell secretion of regulators of trophoblast invasion and uterine natural killer (uNK) cell recruitment and differentiation), birth weight of young pups lower than controls and smaller litters at birth due to failure in fetal development [122]. Intratracheal exposure to CeO_2_ NPs (0.1 mg 3 times during pregnancy) in pregnant mice impairs lung development in offspring and decreases placental efficiency resulting in birth weights below control levels [123]. Conversely, the administration of nanoceria (60 mg/kg for 16 days) to pregnant mice with induced diabetes reduces oxidative stress due to diabetes and thus reduces the teratogenic effects thereof [124]. Another study with repeated oral exposure to CeO_2_ NPs (from premating to the 4th day of lactation) did not induce marked changes in the reproduction of Sprague-Dawley rats and the development of their pups up to the dose of 1000 mg/kg, taking into account that CeO_2_ NPs are sparsely absorbed in parents or their offspring [125]. Oral exposure during pregnancy would therefore be safer compared to intravenous injection or pulmonary exposure, with a low passage of nanoceria through the intestinal barrier as described previously, and most CeO_2_ NPs are excreted in feces. Studies on the intraperitoneal administration of CeO_2_ particles larger than NPs (<5 µm) to pregnant mice found dose-dependent defects in fetal renal development, which results in kidney damage [126] and alterations in the testicular tissue development of the pups [127].

**Figure 3:**
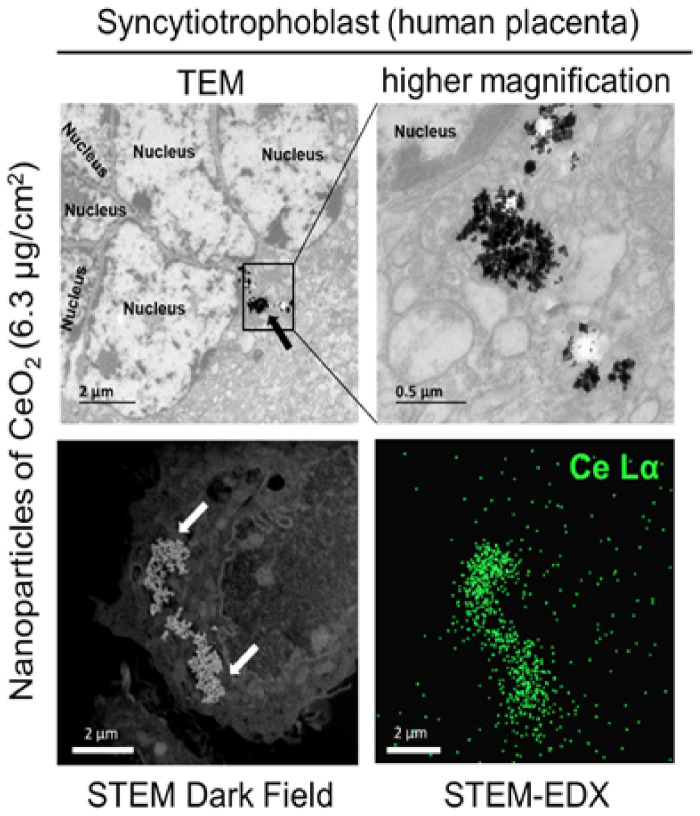
Impacts of nanoceria on term primary culture of human trophoblast. Primary culture of human third trimester cytotrophoblasts exposed for 72 h to CeO_2_ NPs. Observation of CeO_2_ NPs by transmission electron microscopy (TEM), scanning transmission electron microscopy (STEM) combined with energy dispersive X-ray spectroscopy (EDX). Magnified image of TEM is automatically rotated by around 90° to the right. Figure modified after Nedder et al. [117].

Regarding human placenta models, nanoceria have the ability to be internalized by human trophoblasts (both VCTs and ST to the same extent) in primary cultures, with a perinuclear distribution [117] (Figure 3).

**Figure 4:**
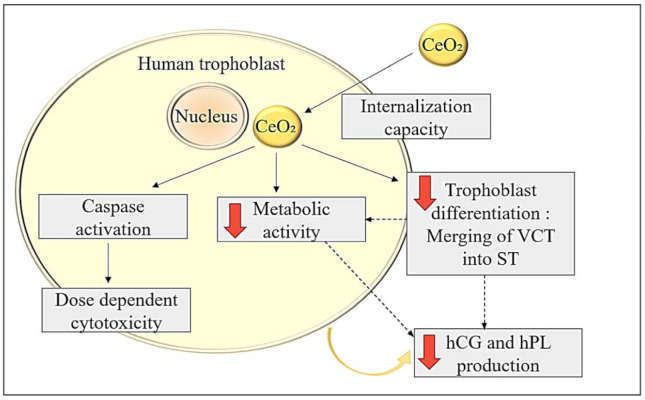
Impacts of nanoceria on term primary human trophoblast cultures. CeO_2_ NPs are internalized in human trophoblasts which exhibit dose-dependent cytotoxicity, with activation of caspases. CeO_2_ NPs decrease the metabolic activity of trophoblasts and also their capacity for differentiation by fusion into a syncytiotrophoblast. The endocrine activity of trophoblasts is disturbed by these CeO_2_ NPs as there is a decrease in the production of hCG and hPL.

Metabolic activity tests revealed that CeO_2_ NPs are cytotoxic to human trophoblasts at high, far-from-physiological concentrations, with a IC50 of 50 µg/cm^2^ at 48 h. This toxicity involves the activation of caspases by CeO_2_ NPs without an increase in oxidative stress. Moreover, even low concentrations of CeO_2_ NPs perturb both morphological and functional differentiation of trophoblasts. Morphologically, the fusion index, which assesses the rate of fusion of VCTs in ST, is reduced when VCTs are exposed to nanoceria, compared to the control. Endocrine function is also altered with a decreased production of hCG and hPL after treatment with CeO_2_ NPs. The decrease in metabolic activity and syncytialization could explain the lower secretion of hormones such as hCG and hPL produced by ST, at non-cytotoxic concentrations of CeO_2_ (Figure 4).

## 4. Discussion

In order to avoid severe effects, as was the case for thalidomide, the safety of a new nanomaterial with promising biomedical applications and already observed environmental exposures must be ensured for pregnant women and the fetus, both in terms of the effects during pregnancy and the long-term effects during development into adulthood. Although CeO_2_ NPs are currently being studied for a large range of applications, notably biomedical, current knowledge does not allow us to conclude that nanoceria have no harmful effects on human pregnancy. In addition, the current exposure of pregnant women to nanoceria through ambient air pollution must be assessed by considering exposure to CeO_2_ NPs mixed with other air pollutants from common emission sources. Fetotoxicity has been found in rodent models, but varies according to the mode of administration of the NPs. The mechanisms underlying this toxicity are not completely defined. However, rodent models cannot easily be extrapolated to determine the impact of nanoceria during human pregnancy. Studies employing human models derived from placental tissues should continue. Comparing results from the different models of human placenta described in this review will ultimately enable a better view and understanding of the mechanisms of action of nanoceria on the placental barrier and the risk for pregnancy outcomes. To date, we lack data regarding the quantity of CeO_2_ NPs present in the human placenta during pregnancy, at term and in the umbilical cord blood or meconium of the newborn. Such data are needed to correlate in vitro findings from placenta models with physiologically relevant doses. This will contribute to a better understanding of the relationship between environmental exposure to CeO_2_ and placental dysfunction with its implications for pregnancy outcomes.

## Figures and Tables

**Table 1:** Summary of the different models to study toxic effects on the human placental barrier.

Models	Interests in Toxicology Studies	Advantages	Drawbacks
Animal models	• impact on pregnancy and outcomes • fetotoxicity studies	*•* in vivo • low cost • chronic exposure possible	• cautious extrapolation to animal model in view of the specificity of human placentation
Ex-vivo placental perfusion	• transplacental passage • placental kinetics and metabolism • placental accumulation of pollutants	• access to organized placental tissue (a whole cotyledon perfused)	• only possible in term placentas • do not allow chronic exposure • nonplacental pharmacokinetic factors
Chorionic villous explant cultures	• barrier permeability and tissular accumulation of pollutants • impact on cell viability • hormonal production	• physiological villi • near-physiological 3D microenvironment	• in vitro • fast ST necrosis • limited time exposures (less than 15 days)
Primary human trophoblast cultures	• impact on trophoblast viability • hormonal production • cellular internalization of pollutants	• recapitulate physiological differentiation to form the syncytium • isolation from term and first trimester placentas	*•* in vitro • limited period of culture due to cell necrosis • not adapted for chronic exposure
Cell line cultures	• impact on cell viability • cellular internalization of pollutants • cell signaling and hormonal production	• low cost • acquired resistance to apoptosis • possible adaptation to long term exposures	• in vitro • cancerous/immortalized cells’ properties distinct from physiological trophoblasts
2D co-cultures and placenta-on-a-chip	• barrier permeability and bypassing • impact on cells’ viability • cell signaling and hormonal production	• near-physiological 3D microenvironment	*•* in vitro • cancerous/immortalized cells’ properties distinct from physiological trophoblasts
3D models (organoids)	still under development	• recapitulate the human placenta villi • anatomically and functionally close to the villous placenta • long term culture possible (chronic exposure possible)	• in vitro • from first trimester placentas only • the polarity of the organoids (ST within the organoid cavity) needs to be reversed for toxicological studies.

**Table 2:** The main co-culture models (reproduced after Nishiguchi et al. [58], Aengenheister et al. [56], Blundell et al. [65]).

Model	Sandwich Culture	Transwell Insert	Placenta-on-a-Chip System
Authors	Nishiguchi et al. 2019 [58]	Aengenheister et al., 2018 [56]	Blundell et al., 2018 [65]
		http://creativecommons.org/licenses/by/4.0/, accessed on 10 November 2021	
Villous cytotrophoblasts	primary VCTs (third trimester) with collagen and laminin coating	BeWo b30	BeWo b30
Villous endothelial cells	human umbilical vein endothelial cells (HUVECs) with fibronectin and gelatin coating	microvascular human placental venous endothelial cell line (HPEC-A2)	human primary placental villous endothelial cells (HPVECs)
Villous mesenchymal fibroblasts	primary human villous mesenchymal fibroblasts (HVMFs) with fibronectin and gelatin coating	none	none
Technology	bottom-up approach using ECM (extracellular matrix) nanofilms	polycarbonate Transwell insert	upper and lower microchannels separated by a thin, semipermeable membrane
Description of the model	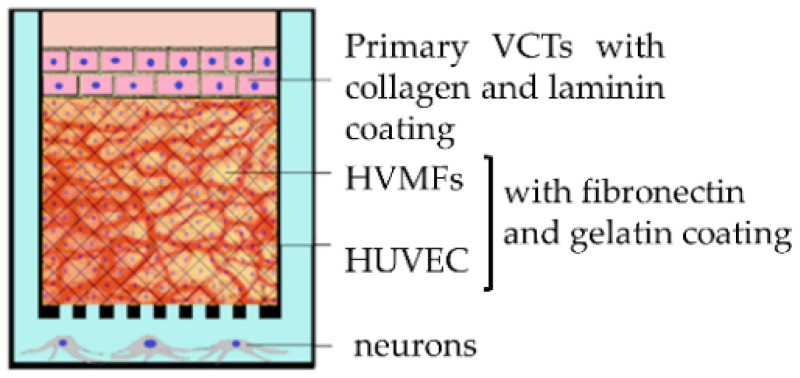	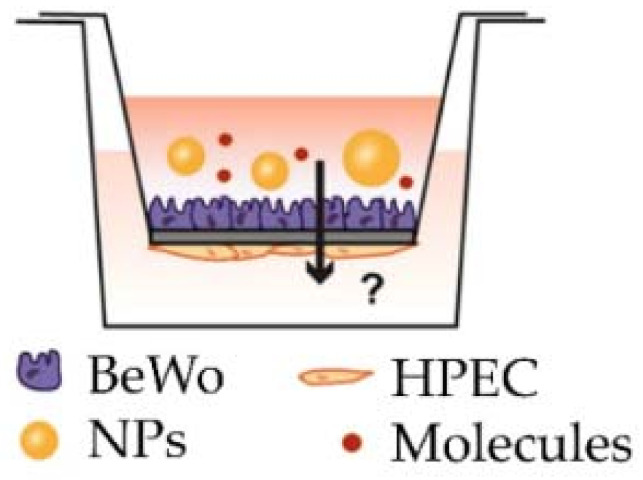	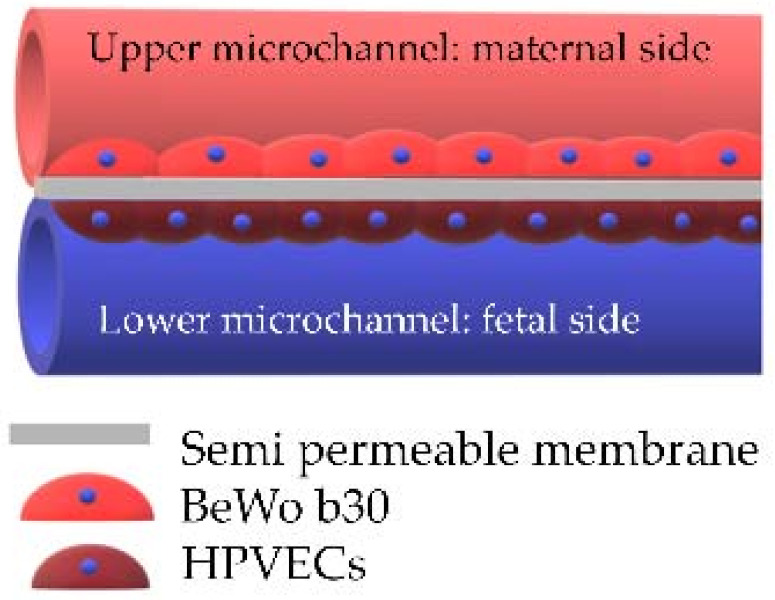

**Table 3:** 3D models (reproduced after Muoth et al. [66] and Turco et al. [67]).

Model	3D Spheroids	Organoids
Author	Muoth 2016	Turco 2018
Villous Cytotrophoblast	BeWo b30 and HTR-8/SVneo	Primary first trimester (8 to 11 WA) proliferative trophoblasts
Villous mesenchymal fibroblasts	Primary human villous mesenchymal fibroblasts (HVMF)	none
Technology	Scaffold-free hanging drop technology (GravityPLUS plates)	Isolation of first trimester proliferative trophoblasts seeded in drops of matrigel in a basal culture medium for the formation of organoids, including growth factors and inhibitors
Description of the model	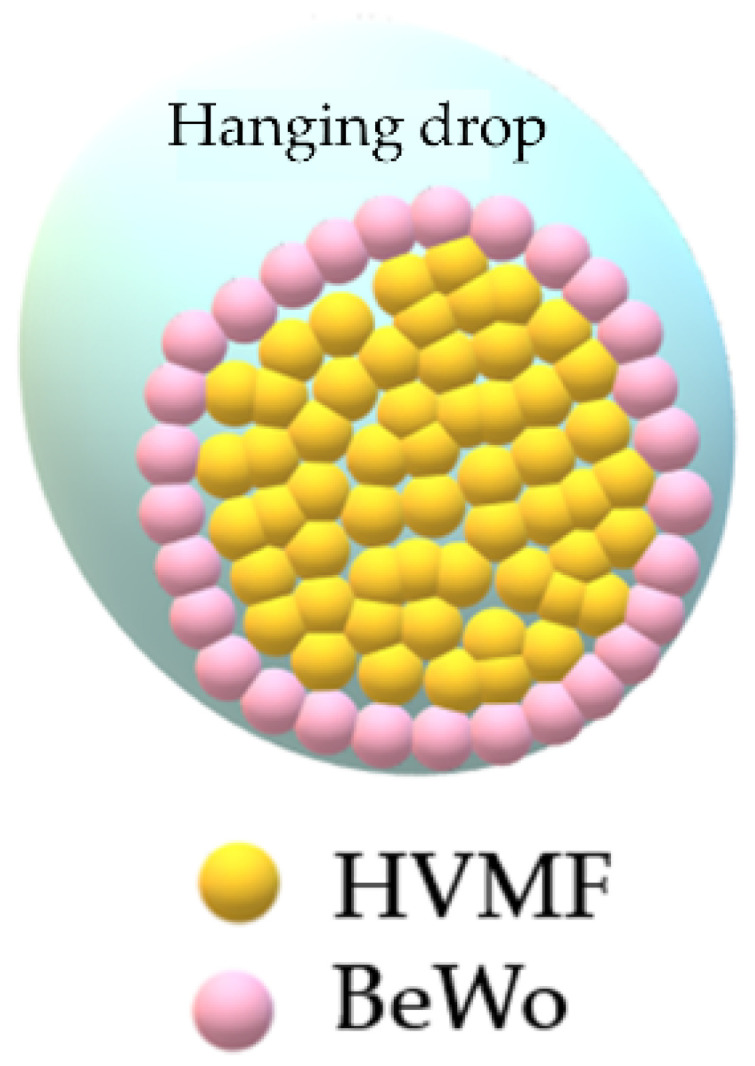	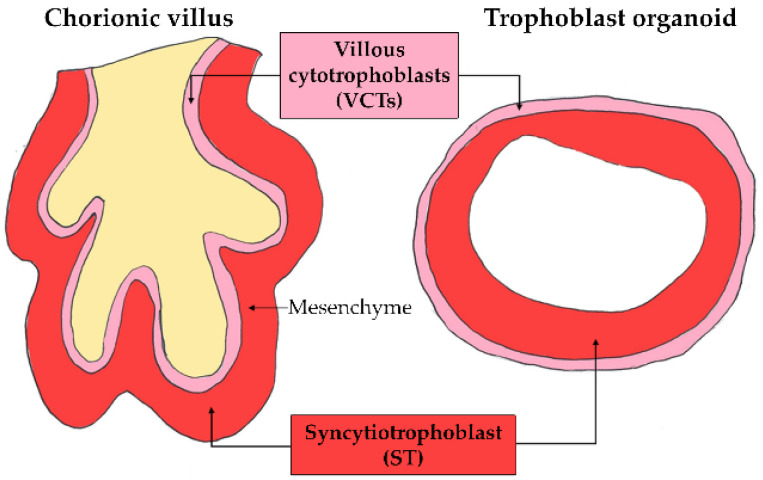

**Table 4:** Different redox reactions of cerium (ROS in red).

Ce^3+^ → Ce^4+^			
Oxidation of Ce^3+^	**O_2_ + Ce^3+^**	**→**	O_2_^•–^ + Ce^4+^
	^•^OH + Ce^3+^	→	OH^−^ + Ce^4+^
			OH^−^ + H^+^ →H_2_O
Superoxide Dismutase (SOD) mimetic activity	O_2_^•–^ + 2H^+^ + Ce^3+^	→	H_2_O_2_ + Ce^4+^
Fenton-like reaction	H_2_O_2_ + Ce^3+^	→	^•^OH + OH^−^ + Ce^4+^
Catalase (CAT) mimetic activity	H_2_O_2_ + 2H^+^ + 2Ce^3+^	→	2H_2_O + 2Ce^4+^
**Ce^4+^ → Ce** **^3+^**			
Reduction of Ce^4+^	H_2_O_2_ + Ce^4+^	→	H^+^ + HO_2_ + Ce^3+^
Superoxide Dismutase (SOD) mimetic activity	O_2_^•–^ + Ce^4+^	→	O_2_ + Ce^3+^
Catalase (CAT) mimetic activity	H_2_O_2_ + 2Ce^4+^	→	2H^+^ + O_2_ + 2Ce^3+^

**Table 5:** Assessment of the impacts of nanoceria on pregnancy.

Data Sources	Model Used	Nanoceria Effects	Type of Nanoceria	Dose and Time Exposure
Nedder et al. 2020	Primary VCTs from human placentas at term of pregnancy	Internalization in both VCT and ST Dose and time dependent cytotoxicity Decrease in differentiation to form the ST Disrupted hormonal production Caspase activation	NM-212 (Joint Research Center nomenclature) polyhedral 28.4 ± 10.4 nm aggregate size 503 ± 55 nm	from 0.1 to 101 µg/cm^2^ until 72 h
Zhong et al. 2020	BALB/c mice	Altered decidualization: disruption of decidual cell secretion of regulators of trophoblast invasion, altered uterine natural killer (uNK) cell recruitment and differentiation Decrease in birth weight Smaller litters because of failure in the fetus development	3−5 nm	5 mg/kg intravenous once a day at on D5, D6 and D7
Paul et al. 2017	C57BL6/J mice	Long-lasting impairment of lung development of the offspring Significant decrease in vascular endothelial growth factor (VEGF) mRNA and protein levels in amniotic fluid and pup lungs Significant decrease in fetal weight and placental efficiency	spherical shape 22.4 ± 0.2 nm aggregate size >1000 nm	intratracheal instillation of 300 µg (100 µg by week) on pregnant mice
Vafaei-Pour et al. 2018	Swiss albino mice with diabetes induced by one dose of intraperitoneal injection of streptozotocin (60 mg/kg)	Reverse the elevation of oxidative stress markers induced by diabetes Diabetes-induced malformation in visceral and spinal of embryo partially restored	no data	60 mg/kg for 16 days
Lee et al. 2020	Sprague-Dawley rats	Cerium was not detected in either parental or pup tissues, not systemically absorbed in parental animals or their pups	polyhedral 14.2 ± 5.0 nm	100, 300 and 1000 mg/kg orally administered during premating, mating, gestation and early lactation periods

## Data Availability

Not applicable.
